# Computational analysis of bacterial RNA-Seq data

**DOI:** 10.1093/nar/gkt444

**Published:** 2013-05-28

**Authors:** Ryan McClure, Divya Balasubramanian, Yan Sun, Maksym Bobrovskyy, Paul Sumby, Caroline A. Genco, Carin K. Vanderpool, Brian Tjaden

**Affiliations:** ^1^Department of Microbiology, Boston University School of Medicine, Boston, MA 02118, USA, ^2^Department of Medicine, Section of Infectious Diseases, Boston University School of Medicine, Boston, MA 02118, USA, ^3^Department of Microbiology, University of Illinois, Urbana, IL 61801, USA, ^4^Department of Pathology, Center for Molecular and Translational Human Infectious Diseases Research, The Methodist Hospital Research Institute, Houston, TX 77030, USA and ^5^Computer Science Department, Wellesley College, Wellesley, MA 02481, USA

## Abstract

Recent advances in high-throughput RNA sequencing (RNA-seq) have enabled tremendous leaps forward in our understanding of bacterial transcriptomes. However, computational methods for analysis of bacterial transcriptome data have not kept pace with the large and growing data sets generated by RNA-seq technology. Here, we present new algorithms, specific to bacterial gene structures and transcriptomes, for analysis of RNA-seq data. The algorithms are implemented in an open source software system called Rockhopper that supports various stages of bacterial RNA-seq data analysis, including aligning sequencing reads to a genome, constructing transcriptome maps, quantifying transcript abundance, testing for differential gene expression, determining operon structures and visualizing results. We demonstrate the performance of Rockhopper using 2.1 billion sequenced reads from 75 RNA-seq experiments conducted with *Escherichia coli*, *Neisseria gonorrhoeae*, *Salmonella enterica*, *Streptococcus pyogenes* and *Xenorhabdus nematophila*. We find that the transcriptome maps generated by our algorithms are highly accurate when compared with focused experimental data from *E. coli* and *N. gonorrhoeae*, and we validate our system’s ability to identify novel small RNAs, operons and transcription start sites. Our results suggest that Rockhopper can be used for efficient and accurate analysis of bacterial RNA-seq data, and that it can aid with elucidation of bacterial transcriptomes.

## INTRODUCTION

Transcriptome assays are increasingly being performed by high-throughput RNA sequencing (RNA-seq) methods. As compared with microarrays, high-throughput sequencing technologies have a number of advantages, including single base pair resolution, low background signal, a large dynamic range of expression levels over which transcripts can be detected, higher levels of reproducibility, smaller sample requirements for starting RNA and no limitation in detecting transcripts that do not correspond to a previously sequenced genome (1). High-throughput sequencing technologies, including systems from Illumina, ABI and Roche 454, have been used to conduct bacterial RNA-seq experiments with a wide range of applications (2,3). For example, bacterial RNA-seq experiments have been conducted with protein-bound RNA (4), with size-selected RNA when small RNAs are of interest (5–8), with 5′ end selected primary transcripts, e.g. via selection of RNA carrying a 5′ tri-phosphate group (9), with RNA from both pathogens and hosts for investigations of host-pathogen interactions (10) and with RNA from whole environments in metatranscriptome studies (11–13).

One of the challenges associated with RNA-seq experiments is analysis of the large resulting data sets. There are numerous tools available [reviewed in (14)] that support various aspects of RNA-seq data processing, management and analysis, but most of these tools are designed primarily for eukaryotic RNA-seq data. As bacterial transcriptomes have different characteristics than eukaryotic transcriptomes, analysis of bacterial RNA-seq data faces different challenges than analysis of eukaryotic RNA-seq data. In bacterial genomes, neighboring genes often overlap; therefore, distinguishing the start of one gene transcript from the end of another adds complexity to transcriptome analysis. The prevalence of polycistronic messages further complicates bacterial transcript assembly. Different promoters may drive expression of a gene or operon under different conditions. And gene models for eukaryotic RNA genes are not appropriate for small regulatory RNAs (sRNAs) in bacteria.

**Figure 1.**
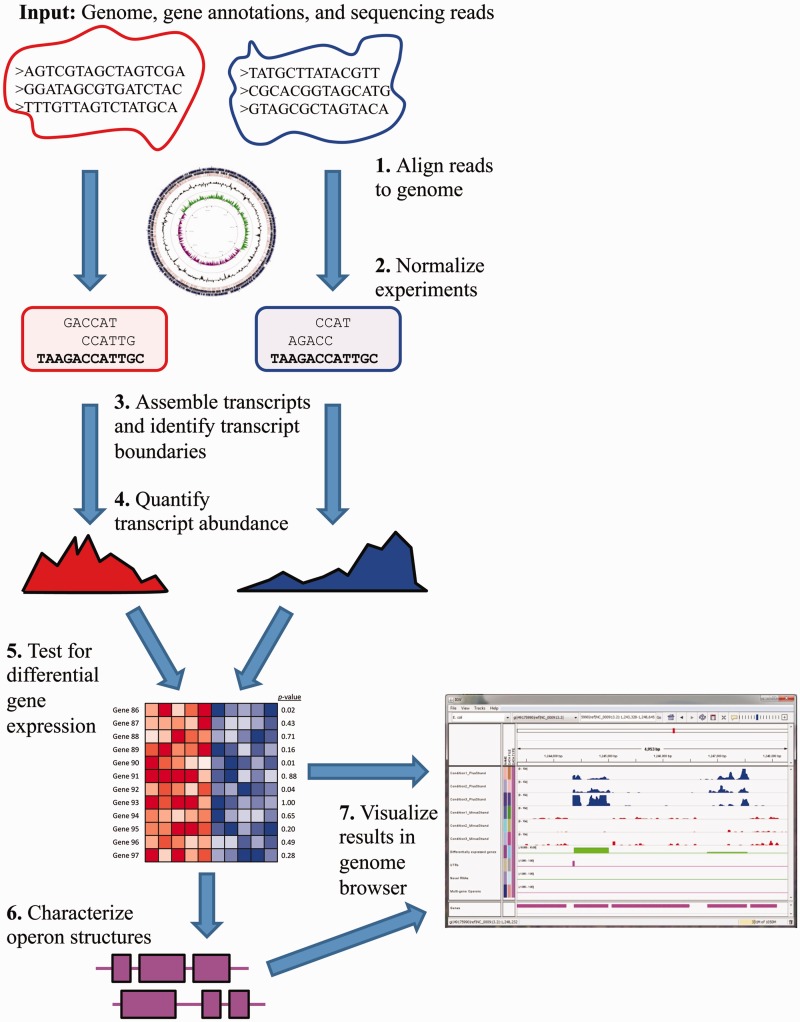
Rockhopper workflow. The input to Rockhopper consists of a genome sequence (FASTA file), gene annotations (PTT and RNT files) and sequencing reads (FASTQ or QSEQ or FASTA files). The different stages of Rockhopper’s workflow are illustrated. Rockhopper’s results are output as tab-delimited text files as well as visually using the Integrated Genomics Viewer.

We present a system and methods designed specifically for analysis of bacterial RNA-seq data. Our system includes new algorithms, which integrate models for sense and antisense sRNAs as well as operon structures, for constructing bacterial transcriptome maps based on RNA-seq data. Starting with sequencing reads output by high-throughput sequencing technology from one or more RNA-seq experiments, our system, called Rockhopper, aligns the reads to a genome, normalizes data from different sequencing experiments, assembles transcripts and identifies transcript boundaries including UTRs and novel small RNAs, quantifies transcript abundance, tests for differential gene expression between experiments, identifies operon structures and enables visualization of the results in a genome browser. The Rockhopper workflow is depicted in Figure 1. The performance of the system is evaluated with data from RNA-seq experiments conducted with five different bacteria and through focused experiments in *Escherichia coli* and *Neisseria gonorrhoeae*. Rockhopper is available at http://cs.wellesley.edu/∼btjaden/Rockhopper.

## MATERIALS AND METHODS

### *Neisseria gonorrhoeae* strain and culture conditions

For experiments involving *N.gonorrhoeae*, strain *N. gonorrhoeae* F62 was used in this study (15). *Neisseria gonorrhoeae* was plated onto GCB agar plates and grown for 16–18 h at 37°C in 5% CO_2_. *Neisseria gonorrhoeae* was then resuspended at a final concentration of 5.0 × 10^7^ CFU/ml in pre-warmed Kerotinocyte Serum Free Media with 0.4 mM CaCl_2_. Cultures were incubated for 2 h at 37°C in 5% CO_2_. RNA was extracted from *N. gonorrhoeae* cultures using TRIzol according to the manufacturer’s instructions.

### *Neisseria gonorrhoeae* RNA-seq experiments

RNA was isolated from *N. gonorrhoeae* F62 as described earlier in the text and DNase treated using TURBO DNase (Ambion) according to the manufacturer’s instructions to remove contaminating DNA. RNA was then ribosomal RNA (rRNA) depleted using the MicrobeEXPRESS kit (Ambion) according to manufacturer’s instructions. A complementary DNA (cDNA) library of the resulting messenger RNA (mRNA) was then prepared using BioChain’s Directional mRNA Sample Prep kit according to the manufacturer’s instructions. Briefly, RNA was fragmented with metal ion scission and treated with PNK. Following PNK treatment, RNA was purified, and 5′ and 3′ Cap and Tail adapters were added. Reverse transcription and 15 cycles of PCR using primers analogous to the adapters generated a cDNA library. This library was then sequenced using 36 or 40 base pair reads on Illumina’s GAIIX machine. As the genome sequence of strain F62 is not available, the sequencing reads were aligned to the genome sequence of *N.gonorrhoeae* FA1090 (16).

### *Escherichia coli* RNA-seq experiments

*Escherichia coli* strains and plasmids used in this study are listed in Supplementary Table S1. *Escherichia coli* strain MG1655 was grown in LB medium to OD_600_∼0.1 at 37°C and exposed to 0.5% α-methylglucoside (αMG) for 15 min. *Escherichia coli* strain CV104 harboring either pHDB3 or pLCV1 plasmids was grown in morpholinepropanesulfonic acid (MOPS) minimal medium supplemented with 0.2% D-glucose to OD_600_∼0.5 and exposed to 0.1 mM IPTG. Total RNA was extracted using the hot phenol method as described previously (17). RNA was treated with TURBO™ DNase (Ambion) according to the manufacturer’s protocol and resolved by electrophoresis on a 1.2% agarose gel to confirm integrity. Library construction and sequencing on the Illumina HiSeq2000 was performed at the W. M. Keck Center for Comparative and Functional Genomics at the University of Illinois at Urbana-Champaign. Ribo-Zero rRNA Removal Meta-Bacteria Kit (Epicentre Biotechnologies) was used to remove rRNA from 1 µg of total RNA. The mRNA-enriched fraction was converted to indexed RNAseq libraries with the ScriptSeq™ v2 RNA-Seq Library Preparation Kit (Epicentre Biotechnologies). The libraries were pooled in equimolar concentration and quantitated by quantitative PCR (qPCR) with the Library Quantification kit Illumina compatible (Kapa Biosystems). The pooled libraries were sequenced for 101 cycles plus 7 cycles for the index read on a HiSeq2000 using TruSeq SBS version 3 reagents. The fastq files were generated with Casava 1.8.2 (Illumina).

### *Salmonella enterica* RNA-seq experiments

The *S. enterica* serovar Typhimurium LT2 (*Salmonella*) wild-type or a mutant Salmonella strain carrying a deletion of the sRNA, sgrS, (DB111) was grown in minimal MOPS medium (Teknova) to mid-logarithmic phase. Total RNA was extracted using hot phenol (17) and was treated with Turbo DNase (Ambion). Library generation and sequencing was performed by the W.M. Keck Center for Comparative and Functional Genomics at the University of Illinois at Urbana-Champaign as described earlier in the text.

### *Streptococcus pyogenes* RNA-seq experiments

The *S. pyogenes* strain MGAS2221 was grown in Todd–Hewitt broth with 0.2% yeast extract to both the exponential and stationary phases of growth. RNA was isolated from exponential and stationary phase aliquots via a mechanical disruption method as previously described (18). Contaminating DNA was removed from the isolated RNA through use of TURBO DNase (Ambion) as per the manufacturer’s instructions. Purified RNA samples were split, with different rRNA depletion methods performed on each split sample. One rRNA depletion method made use of the Ribo-Zero rRNA Removal Kit (Epicentre Biotechnologies), the other rRNA depletion method made use of Terminator 5′-phosphate-Dependent Exonuclease (Epicentre Biotechnologies), in both cases the reagents were used as per the manufacturer’s instructions. The rRNA-depleted RNA samples were used to generate cDNA libraries using the ScriptSeq mRNA-Seq Library Preparation Kit (Epicentre Biotechnologies) as per the manufacturer’s instructions. Briefly, RNA treated with the Ribo-Zero kit was fragmented with metal ion scission, whereas Terminator 5′-phosphate-Dependent Exonuclease-treated RNA was not fragmented. All RNAs were used in cDNA synthesis reactions with tagged random hexamers, RNA was hydrolyzed, a tag was added to the 3′ ends of the cDNAs and the cDNAs were then used in limited cycle (15 cycles) PCR reactions with primers analogous to the tags at the 5′ and 3′ ends of the generated cDNAs. PCR-generated DNA was size selected (150–250 bp) via agarose gel electrophoresis and sequenced using 72 nucleotide reads on Illumina’s GAIIx machine.

### *Xenorhabdus nematophila* RNA-seq experiments

Details of the *X. nematophila *RNA-seq experiments have been published previously (19). In summary, data were collected from four size-selected RNA-seq experiments using *X. nematophila* wild-type and *rpoS* mutant strains (20). Before sequencing, rRNAs and transfer RNAs were targeted for depletion, and samples were size-selected for RNAs 18–200 nucleotides in length to enrich for small RNAs.

### Simulated RNA-seq experiment

To assess the accuracy of Rockhopper’s transcript abundance estimates and the specificity of Rockhopper’s novel transcript identifications, we simulated an RNA-Seq experiment in *N. gonorrhoeae* using the Flux Simulator (http://flux.sammeth.net), which models RNA-seq experiments *in silico*. First, using the Flux Simulator, we simulated an artificial expression profile based on the 2002 annotated genes in the *N. gonorrhoeae* genome (21). The Flux Simulator chooses gene expression levels at random based on a mixed power and exponential law. We used a mean of 25 for the exponential distribution used to model variations in transcription start sites for each gene, we used random hexamers as primers for first strand synthesis during reverse transcription, and we used uniform random fragmentation during the Flux Simulator’s fragmentation process. The Flux Simulator’s default values were used for PCR amplification during final library preparation. Finally, we used the Flux Simulator to simulate 31 million single-end reads of length 72 nt. Rockhopper aligned to the *N.gonorrhoeae* genome ∼15 million of the sequencing reads produced by the Flux Simulator. We used these aligned reads together with the simulated expression profiles for the annotated *N.gonorrhoeae* genes to assess Rockhopper’s accuracy in quantifying transcript abundances and to evaluate Rockhopper’s propensity for false positive identifications of novel transcripts.

### *Neisseria gonorrhoeae* primer extension

For primer extension, 10 μg of bacterial RNA was incubated with a [α-^32^P]ATP radiolabeled oligonucleotide probe at varying temperatures corresponding to the probe’s melting temperature. Probes (Supplementary Table S2) were designed to be 100 bp downstream of transcriptional start sites as determined by RNA-seq and were labeled using T4 PNK for 60 min at 37°C followed by 2 min at 90°C to inactivate the PNK. Following probe hybridization to bacterial RNA, the probes were extended using reverse transcriptase for 1 h at 41°C. Single-stranded DNA products were then run through an 8% TBE–Urea gel along with a single-stranded DNA ladder, which was also radiolabeled. Size of primer extension products was determined by plotting the base 10 logarithm of the size of each ladder marker against distance traveled in the gel on semi-log paper. Primer extension products were then sized using the resulting standard curve.

### *Neisseria gonorrhoeae* quantitative reverse transcriptase-PCR

Quantitative reverse transcriptase-PCR (qRT-PCR) was carried out using the One-Step QuantiTect SYBR green RT-PCR kit (Qiagen) on an ABI Prism 7700 sequence detection system (Applied Biosystems, Foster City, CA). A total of 125 ng of RNA was used in each reaction mixture along with 2 ng of each primer (Supplementary Table S2). The relative mRNA levels were evaluated using the comparative cycle threshold (ΔΔCT) method. The relative expression level of each gene was normalized to the endogenous NGO0616 gene and is represented as the ratio to that gene.

### *Neisseria gonorrhoeae* RT-PCR of operons

For operon prediction, primers (Supplementary Table S2) were designed to hybridize to the beginning of the upstream gene and the end of the downstream gene so that the entire two-gene operon was amplified by RT-PCR, not merely a part of it. RT-PCR was carried out using 125 ng of bacterial RNA and 2 ng of primers. Products were then run out on a 1.0% agarose gel with a double-stranded DNA ladder.

### *Escherichia coli* 5' RACE

A 5′ RACE was performed as described previously (22). Briefly, RNA was extracted using hot phenol (17) from *E.coli* MG1655 cells grown to mid-logarithmic phase in minimal MOPS medium with 0.2% fructose. RNA was then subjected to DNase (Ambion) treatment. Half of each DNase-treated RNA sample was treated with Tobacco Acid Pyrophosphatase (Epicentre Biotechnologies), and the other half were left untreated. An RNA oligonucleotide adapter (O-DB236) was ligated to both Tobacco Acid Pyrophosphatase-treated and untreated RNA samples. Oligonucleotides specific to genes of interest (*lpp:* O-DBRT237, *hns: *O-DBRT245, *ppa:* O-DBRT257, *thrS:* O-DBRT261, *sodA:* O-DBRT269, *cspE:* O-DBRT253, *panD:* O-DBRT265) were used in reverse transcription reactions (enzyme from Invitrogen) to generate cDNA. Lastly, oligonucleotides complementary to the RNA adapter sequence (O-DB282) and nested oligonucleotides specific to genes (*lpp:* O-DB238, *hns:* O-DB246, *ppa:* O-DB258, *thrS:* O-DBRT262, *sodA:* O-DBRT270, *cspE:* O-DBRT254, *panD:* O-DBRT266) were used to amplify PCR products, which were subsequently cloned into a TOPO vector (Invitrogen). Multiple clones were sequenced with oligonucleotides (*lpp:* O-DBseq240, *hns:* O-DBseq248, *ppa:* O-DBseq260, *thrS:* O-DBseq264, *sodA:* O-DBseq272, *cspE:* O-DBseq256, *panD:* O-DBseq268) to identify transcription initiation sites. Oligonucleotides are listed in Supplementary Table S3.

### *Escherichia coli* RT-qPCR

*Escherichia coli* strain MG1655 was grown in LB medium to OD_600_∼0.1 at 37°C and exposed to 0.5% αMG for 15 min. Total RNA was extracted using the hot phenol method as described previously (17). RNA was treated with TURBO™ DNase (Ambion) according to the manufacturer’s protocol and resolved by electrophoresis on a 1.2% agarose gel to confirm integrity. RNA concentrations were determined spectrophotometrically. cDNA was generated using SuperScript™ III Reverse Transcriptase (Invitrogen) according to the manufacturer’s protocol using random hexamer primers (Applied Biosystems). The qPCR was performed using SYBR green (Applied Biosystems) PCR master mix in the Mastercycler ep realplex (Eppendorf) thermocycler (primers listed in Supplementary Table S4). The relative quantitation method was used to calculate relative change in gene expression between the samples. The relative mRNA levels were normalized to a housekeeping gene *rrsA* encoding 16S rRNA in *E. coli*.

### *Escherichia coli* RT-PCR

*Escherichia coli* K12 wild-type cells (MG1655) were grown in minimal MOPS medium (Teknova) supplemented with 0.4% glycerol at 37°C and subcultured 1:200 in fresh medium. The cultures were then grown to an optical density at 600 nm (OD_600_) of ∼0.3. Total RNA was extracted by the hot-phenol method (23). RT was performed with 500 ng of DNA-free (Ambion) DNase-treated RNA using the SuperScript III reverse transcriptase (Invitrogen). The oligonucleotides used for the reverse transcription procedure are listed in Supplementary Table S5. The cDNA products generated from the reverse transcription were then used as the templates for PCR amplification using the GoTaq (Promega) PCR system. The oligonucleotides used for the PCR amplification are listed in Supplementary Table S6.

### Aligning reads to genome

Rockhopper’s approach for aligning reads to a reference genome follows that of Bowtie2 (24). An FM-index (25) is created for the genome based on the Burrows–Wheeler transform (26). After index creation, a read of length *m* can be aligned exactly to a genome of size *n* in *O*(*m*) time with small constants. When a read does not align exactly to a genome, seed regions of the read are aligned to the genome, and the seed alignments are extended with a dynamic program using the Smith–Waterman algorithm (27). To maintain the efficiency of the approach, i.e. aligning a read of length *m* in *O*(*m*) computation, the entire *m* × *m* dynamic programming table is not populated but rather entries within *k* of the diagonal, where *k* is a parameter representing the maximum number of allowed mismatches, insertions or deletions in the alignment (*k* is 15% of the read length by default). Mismatch, insertion and deletion scores in the dynamic programming table are based on Phred quality scores (28,29), which represent the error probability of each sequenced nucleotide in each read. The dynamic programming alignment determines the optimal alignment, or multiple alignments if there is more than one optima, with respect to the error scores subject to *k*. To improve efficiency for aligning large numbers of reads, Rockhopper is fully parallelizable; Rockhopper self-identifies the number of processors on the machine on which it is executing and distributes its computation across the available processors.

### Identifying transcript boundaries

To generate a transcriptome map based on reads from an RNA-seq experiment, a multi-step approach is used. First, a set of transcript seeds is identified corresponding to annotated genes and to novel transcript seeds. Novel transcript seeds are genomic regions at least *w* nucleotides in length (*w* is 10 by default) such that every nucleotide in the region has at least *T* reads mapping to the nucleotide, where the threshold *T* is a function of the average number of reads per nucleotide throughout the genome. Novel transcript seeds are maximal, i.e. the number of reads mapping to the nucleotide immediately upstream and to the nucleotide immediately downstream of a novel transcript seed is less than *T*. Transcript seeds correspond to genomic regions rather than RNA transcripts.

Each transcript seed is then extended, upstream and downstream, using a Bayesian approach. Let *s* refer to a transcript seed and *r* be a genomic region consisting of one or more nucleotides adjacent to *s*. Our goal is to determine whether *r* corresponds to part of the same transcript as *s*, i.e. whether *s* should be extended to include *r*. Using Bayes’ theorem, we have

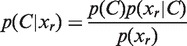

where *x_r_* is the number of reads mapping to *r* and *C* is a dependent class variable with two outcomes, *C* = {*c_r_*_↔_*_s_*, *c_r_*_|_*_s_*} with *c_r_*_↔_*_s_* corresponding to *r* and *s* being co-transcribed and *c_r_*_|_*_s_* corresponding to *r* and *s* not being co-transcribed. Following others (30–32), the probability *p*(*x_r_* | *c_r_*_↔_*_s_*) is determined by fitting a Poisson distribution to the number of reads mapping to *s*, based on the assumption that reads are sampled uniformly and independently. The probability *p*(*x_r_* | *c_r_*_|_*_s_*) is determined from a background geometric distribution based on the number of reads mapping antisense throughout the genome to annotated protein coding genes. Using the maximum a posteriori estimate, the seed *s* is extended to include *r*, or not, based on

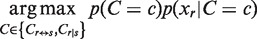

which is equivalent to the maximum likelihood estimate, as uniform prior probabilities are assumed. Finally, after each transcript seed has been extended, adjacent or overlapping transcript seeds are merged if the distributions of reads across two adjacent or overlapping transcript seeds are significantly similar. The transcriptome map reported by Rockhopper corresponds to the set of merged, extended transcript seeds.

### Differential gene expression

Rockhopper computes a *P*-value for the differential expression of each gene using the approach of Anders and Huber (33). For a given gene, let *R*_1_ and *R*_2_ refer to the sum of normalized reads aligning to the gene across all replicates in condition 1 and condition 2, respectively. Let the probability of the event *R*_1_ = *x* and *R*_2_ = *y* be denoted as *p*(*x*, *y*). Then, the *P*-value for a pair of observed read summations, *R*_1_ and *R*_2_, is the sum of probabilities less than or equal to *p*(*R*_1_, *R*_2_) and can be expressed as

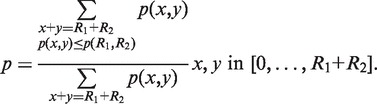



To compute *p*(*x*, *y*), assuming independence of conditions 1 and 2, we calculate the product of the probabilities that *R*_1_ = *x* and *R*_2_ = *y* by approximating the random variables *R*_1_ and *R*_2_ with negative binomial distributions whose variance parameters are estimated as described earlier in the text. From the resulting *P*-values, differentially expressed genes are determined by computing *q*-values based on Benjamini–Hochberg correction (34) with a false discovery rate <1%.

### Operon prediction

To estimate the likelihood that consecutive genes on the same strand are part of the same operon, a naïve Bayes classifier is used based on prior operon probabilities and on two features, intergenic distance and correlation of gene expression across RNA-seq experiments. The prior probability that two consecutive genes on the same strand are co-transcribed is estimated as 1—(Number of directons/Number of pairs of genes on the same strand), where a *directon* is a set of consecutive genes on the same strand (35). As examples, we estimate the prior probability of co-transcription for two consecutive genes on the same strand as 83% for *S.pyogenes*, 74% for *N.gonorrhoeae* and 73% for *E.coli*. Given the distance between two consecutive genes, the probability that they correspond to the same operon is based on the distribution of distances between consecutive genes on the same strand, smoothed via an Epanechnikov kernel and the probability that they do not correspond to the same operon is based on the distribution of distances between consecutive genes on the opposite strand, smoothed via an Epanechnikov kernel (Supplementary Figure S1). The probability that two consecutive genes are similarly expressed is based on their correlation of expression across the RNA-seq experiments.

## RESULTS

### RNA-seq experiments

Data were gathered from 75 RNA-seq experiments conducted in five different bacteria: *E. coli*, *N.gonorrhoeae*, *S. enterica*, *S. pyogenes* and *X. nematophila*. Among the 75 experiments, 61 experiments used protocols that maintained strand specificity of the sequencing reads, five experiments used size selection to focus on sRNAs and the length of the sequencing reads ranged in the experiments from 36 to 100 nt. Details of the RNA-seq experiments including GEO accession numbers for access to the data files are provided in Supplementary Table S7. Altogether, the RNA-seq experiments yielded over two billion sequencing reads corresponding to 189 billion nt. The outputs from each experiment are sequencing reads along with an error probability for each nucleotide in each sequencing read (in a Fastq or QSEQ file), and this output constitutes the input to Rockhopper’s bacterial RNA-seq data analysis.

### Aligning reads to a reference genome

The first step in processing RNA-seq data normally involves aligning the reads to a genome, when a reference genome is available. Numerous mature tools exist for aligning reads to a genome [reviewed in (36)]. Most commonly, read alignment tools are based on methods that index the genome using an auxiliary data structure such as a hash table, suffix tree, enhanced suffix area or FM-index. The approach used by Rockhopper for aligning reads to a genome is similar to that of Bowtie2 (24). A Burrows–Wheeler index (26) based on the full-text minute space (25) is created for the reference genome. After creating the index, for each read, an exact alignment to the genome (index) is attempted, and, if unsuccessful, an inexact alignment to the genome is attempted by aligning seed regions from the read to the genome and extending the seed alignments with a dynamic program. For inexact alignments, a quality aware scoring function based on the error probability of each sequencing read nucleotide is used to ensure that the highest quality alignment is found for a read.

**Figure 2.**
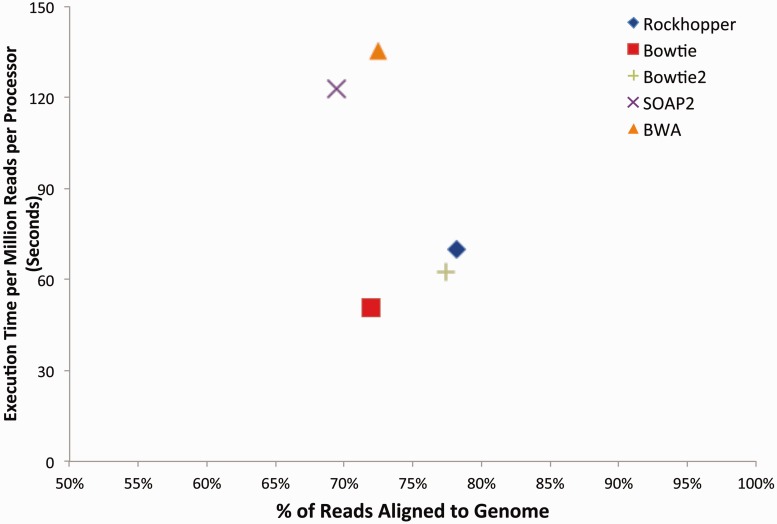
Aligning sequencing reads to a genome. The performance of five tools for aligning reads to a genome is shown. The five tools are Rockhopper (version 1.00), Bowtie (version 0.12.7), Bowtie2 (version 2.0.0-beta5), SOAP2 (version 2.21) and BWA (version 0.6.2). Each tool is based on an FM-index, and each tool was executed on the same machine with default parameters using the same number of processors. The tools were evaluated by the percentage of 2 134 636 656 reads that they successfully aligned to a reference genome (*x*-axis) and by the execution time they required per million reads per processor (*y*-axis). The reads come from 75 RNA-seq experiments conducted using five different bacteria.

To evaluate Rockhopper’s ability to align reads to a genome, we compared its performance with that of several leading sequence alignment tools: SOAP2 (37), BWA (38), Bowtie (39) and Bowtie2 (24). Details of each tool’s performance on each of the 75 RNA-seq data sets are provided in Supplementary Table S7. In summary, among the tools, the percentage of the ∼2.1 billion reads that were successfully aligned ranged from 69 to 78%, and the execution time per million reads per processor ranged from 51 to 135 s (Figure 2). Rockhopper had the highest accuracy in aligning reads to a genome, and it was the third fastest tool, behind Bowtie and Bowtie2, suggesting that Rockhopper is competitive with the leading tools for aligning reads.

### Normalization

To allow for comparison of data from different samples and experiments, each RNA-seq data set must be normalized. Most commonly, data from each sample is normalized by the total read count. For instance, total read counts are used for normalization when reads per kilo base per million mapped sequence reads (RPKM) values are used as gene expression measures (30). However, total read count normalization can be problematic, as a few highly expressed genes often account for the majority of total read counts, and there is no guarantee that these few highly expressed genes have similar levels of expression across different biological conditions (40). As shown in Supplementary Figure S2, for 10 samples, two each from five different bacteria, all depleted for rRNA before sequencing, the 5% of most expressed genes account for between 55 and 98% of total read counts in the samples.

Alternatively, expression levels of single housekeeping genes can be used for normalization, a standard technique for normalizing qRT-PCR expression measures. However, this method, too, may be problematic in that it can be difficult to identify genes with consistent and stable expression (40). Instead, Rockhopper normalizes read counts for each sample by the upper quartile gene expression level after excluding genes with zero expression (zero reads mapping to the gene). Bullard *et al.* (40) show that, among normalization techniques they investigated, upper quartile normalization had the best concordance with qRT-PCR data.

### Assembling transcripts and identifying transcript boundaries

Bacterial genome annotations typically indicate translation boundaries (start and stop sites) for most protein coding genes and transcription boundaries for many RNA genes, primarily rRNAs and transfer RNAs. However, annotations typically lack transcription boundaries for protein-coding genes, and they lack annotations for many small non-coding RNA genes. Here, we present a novel approach for constructing a bacterial transcriptome map consisting of the location of every transcript evinced by a set of RNA-seq experiments. The transcriptome map is determined, first, by identifying a set of transcript seeds consisting of annotated genes and high confidence novel transcripts corresponding to genomic regions where a significant number of reads were found to align. The transcript seeds are then extended using a Bayesian approach to identify more precise transcript boundaries (see ‘Materials and Methods’ section). Transcriptome maps were generated from RNA-seq data for five different bacteria (Supplementary Table S8).

**Figure 3.**
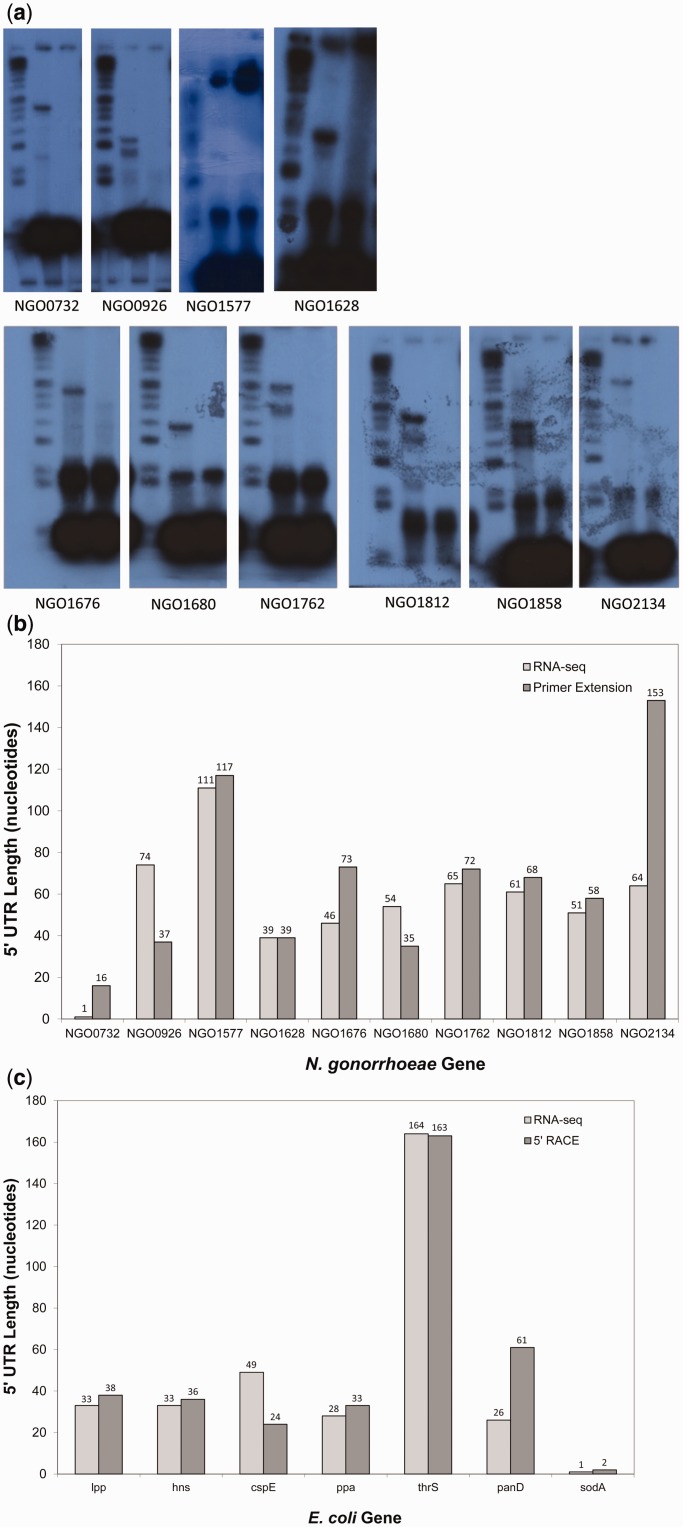
5′ UTR analysis. (**a**) Results from primer extension for 10 *N. gonorrhoeae* genes. Probes were designed to lay down 100 nucleotides upstream of the transcription start site identified by analysis of the RNA-seq data. Gene designations correspond to *N. gonorrhoeae *strain FA1090. (**b**) For 10 *N. gonorrhoeae* genes, the length of the 5′ UTR as determined from RNA-seq data is depicted (light gray) and the length of the 5′ UTR as determined from primer extension analysis is depicted (dark gray). Gene designations correspond to *N. gonorrhoeae* strain FA1090. (**c**) For seven *E. coli* genes, the length of the 5′ UTR as determined from RNA-seq data is depicted (light gray) and the length of the 5′ UTR as determined from 5′ RACE is depicted (dark gray).

To evaluate the accuracy of our method in characterizing transcript boundaries, we performed primer extension experiments for 10 randomly chosen genes in *N. gonorrhoeae* that had significant expression in the RNA-seq data (Figure 3a). For the 10 genes, primers were used upstream of each transcription start site predicted by our method based on the *N. gonorrhoeae* RNA-seq data. Figure 3b shows the lengths of 5′ UTRs for the 10 genes as determined by the primer extension results and as identified from the RNA-seq data. For 5 of the 10 genes (NGO1577, NGO1628, NGO1762, NGO1812 and NGO1858), the length of the 5′ UTR identified by our approach is within 7 nt of the length of the 5′ UTR as estimated from the primer extension experiments. For 3 of the 10 genes (NGO0732, NGO1676 and NGO2134), our method based on the RNA-seq data underestimates the length of the 5′ UTR, as compared with the primer extension results, by 15, 27 and 89 nt, respectively. For 2 of the 10 genes (NGO0926 and NGO1680), our method based on the RNA-seq data overestimates the length of the 5' UTR, as compared with the primer extension results, by 37 and 16 nt, respectively.

As further evaluation of our approach for identifying transcript boundaries, we performed 5′ RACE on seven randomly chosen genes in *E. coli* that had significant expression in the RNA-seq data. Figure 3c shows the lengths of 5′ UTRs for the seven genes as determined by 5′ RACE and as identified from the RNA-seq data. For five of the seven genes (*lpp*, *hns*, *ppa*, *thrS* and *sodA*), the length of the 5′ UTR identified by Rockhopper is within 7 nt of the length of the 5′ UTR as determined from 5′ RACE. For one of the seven genes (*cspE*), our method overestimates the length of the 5′ UTR, as compared with the 5′ RACE results, by 25 nt. For one of the seven genes (*panD*), our method underestimates the length of the 5′ UTR, as compared with the 5′ RACE results, by 35 nt.

Altogether, the transcription start site identified by Rockhopper based on RNA-seq corresponded closely (within 7 nt) to the experimentally evinced transcription start site 59% of the time (5 of 10 genes in *N. gonorrhoeae* as determined by primer extension and 5 of 7 genes in *E. coli* as determined by 5′ RACE). For the remaining cases, Rockhopper mis-identified the transcription start site by between 15 and 89 nt. There was no significant correlation between the accuracy of Rockhopper’s transcription start site identifications and gene expression levels or 5′ UTR lengths. We did not evaluate Rockhopper’s ability to identify transcription start sites when using differential RNA-seq (dRNA-seq) data based on 5′ end selection of primary transcripts, which enables more reliable discernment of precise transcription start sites (9).

We proceeded to generate two transcriptome maps based on *N. gonorrhoeae* RNA-seq data, one based on a size-selected RNA-seq experiment and one based on non-size-selected RNA-seq experiments. The two transcriptome maps based on the *N. gonorrhoeae* RNA-seq data include 34 small transcript identifications common to both maps that do not correspond to any RefSeq (21) annotated gene, seven of which are antisense to annotated protein-coding genes. To determine whether any of these small transcript identifications corresponded to previously characterized sRNAs in *N. gonorrhoeae*, we queried the Rfam database (41) and found four *N. gonorrhoeae* sRNAs: tmRNA, 4.5S RNA, RNase_P and NrrF. All four of these sRNAs were among the small transcripts identified in our transcriptome maps. In another study [McClure, Tjaden, and Genco, submitted for publication], seven small transcript candidates were chosen at random from the 34 small transcripts identified by our analysis, and these seven candidates were evaluated by northern blots. Three of the seven candidates are antisense to protein-coding genes. For each of the seven candidates, northern blots showed evidence of a small transcript. These results suggest that our method for generating a transcriptome map based on RNA-seq data is able to identify novel sRNA transcripts.

To estimate a lower bound on false positive novel transcript predictions, we evaluated our method on data from a *computationally simulated* RNA-seq experiment in *N. gonorrhoeae* using Flux Simulator (http://flux.sammeth.net). The simulated RNA-seq experiment generated 31 million reads of length 72 nt based on expression from the annotated genes in *N.gonorrhoeae* using an average transcription start site deviation of 25 nt (see ‘Materials and Methods’ section). The advantage of using a simulated RNA-seq experiment, as opposed to a non-simulated RNA-seq experiment, is that the location and abundance of every transcript is known *a priori*; therefore, the transcriptome map generated by our method can be compared precisely to the set of true (simulated) transcripts. Any novel transcript identifications by our method must be false positives, as all transcripts are known in the simulated experiment. Based on the simulated experiment, Rockhopper generated a transcriptome map consisting of a single novel transcript identification. This lone false positive transcript identification suggests an upper bound on the specificity of our transcriptome map.

### Quantifying transcript abundance

**Figure 4.**
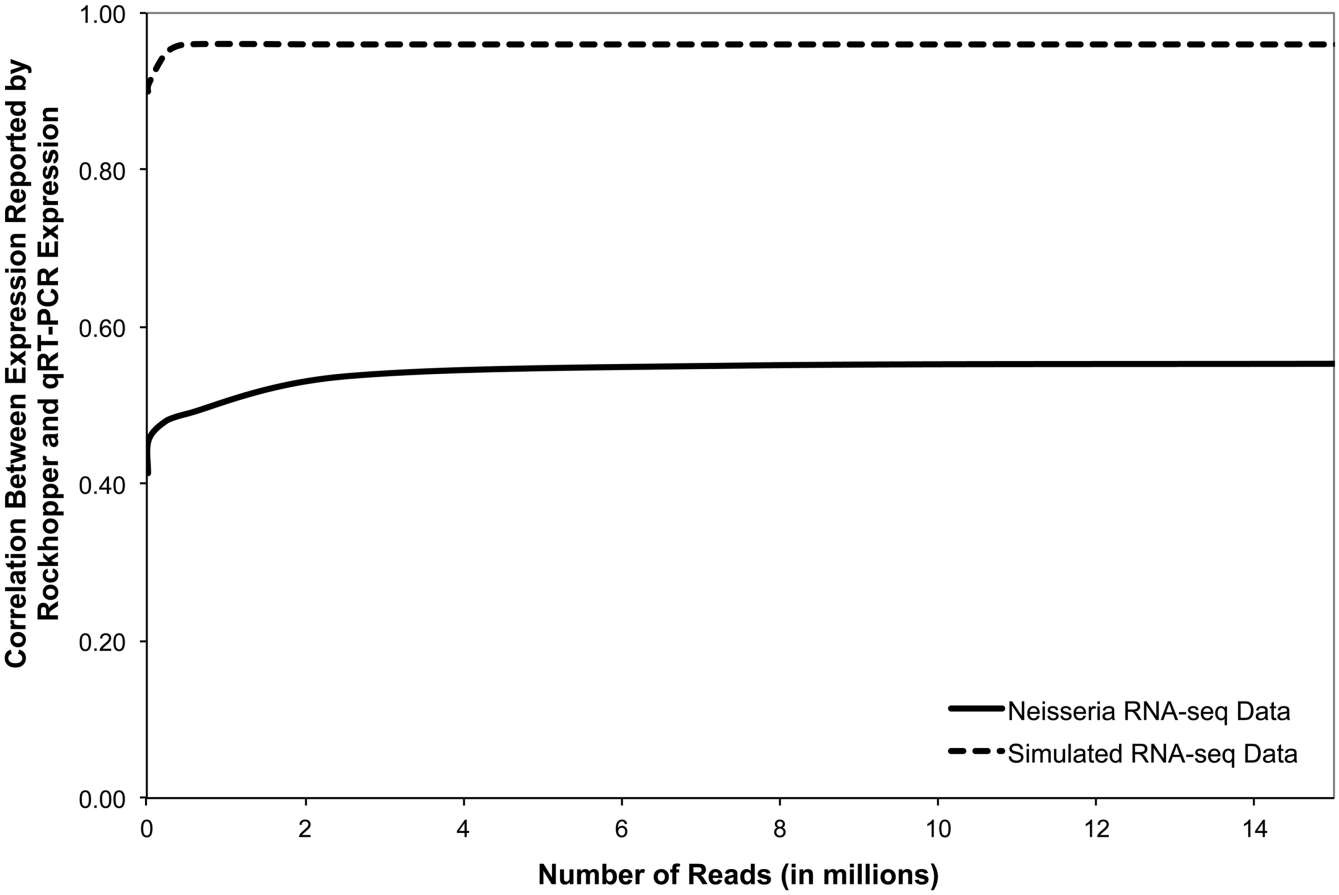
Correlation between expression abundances determined by Rockhopper based on RNA-seq data and confirmed expression abundances. Correlation is computed based on expression abundances determined by Rockhopper when different sized random subsets of RNA-seq reads are used. The solid curve represents, for nine *N. gonorrhoeae* genes, the correlation between expression levels as determined by Rockhopper based on a RNA-seq experiment and as determined via qRT-PCR. The dashed curve represents, for 2002 *N.gonorrhoeae* genes, the correlation between expression levels as determined by Rockhopper based on a simulated RNA-seq experiment and as determined via simulation.

Common measures for quantifying gene expression from RNA-seq experiments are (RPKM) (30) and FPKM (42) that sum the number of reads for a gene and divide by the gene’s length and the total number of reads in the sample, modulo a constant. We use a similar measure except that instead of dividing by the total number of reads in the sample, we divide by the upper quartile of gene expression, excluding genes with zero expression, to increase robustness of the measure (see ‘Normalization’ section earlier in the text). To evaluate the extent to which transcript abundance levels reported by our system correspond to gene expression levels, we compared Rockhopper’s transcript abundance estimates based on RNA-seq data from *N. gonorrhoeae* with gene expression levels determined from qRT-PCR experiments. qRT-PCR was performed for nine *N.gonorrhoeae* genes from three biological replicates. Figure 4 (solid curve) shows, for nine genes, the correlation between Rockhopper’s reported expression levels and those observed via qRT-PCR. When all aligned 15 million sequencing reads from the *N. gonorrhoeae* RNA-seq data are used, the RNA-seq estimated expression levels have a correlation of 0.55 with the qRT-PCR data. We then estimated gene expression levels using different sized subsets of randomly chosen sequencing reads and found that the correlation with qRT-PCR results decreased as smaller subsets of reads were used, but only gradually, as the number of aligned reads decreased from 15 to 2 million.

Similarly, using a computationally simulated RNA-seq experiment with *N. gonorrhoeae*, we evaluated the correlation between Rockhopper’s estimated transcript abundance levels and the known simulated expression levels for all 2002 annotated protein-coding genes. When all simulated reads were used, the estimated gene expression levels had a correlation of 0.96 with the known simulated expression levels (Figure 4, dashed curve). When smaller sized subsets of randomly chosen simulated reads were used to estimate gene expression levels, the correlation decreased but only gradually. These results suggest that our estimated transcript abundance levels are correlated with true gene expression levels, though the extent of correlation is substantially higher in the case of simulated data using 2002 genes than in the case of non-simulated data using nine genes. The results also indicate that for the RNA-seq experiments evaluated, when using only 2 million reads, which is an order of magnitude less than the number of reads available in our experiments, there is not a significant degradation in transcript abundance estimates. As high-throughput sequencing technologies have limits on how many reads they can generate, these findings may be relevant to investigators when deciding how many samples to multiplex in their RNA-seq experiments.

Although the relationship between GC-content and gene expression is unclear (43), it is well-known that transcriptome-sequencing technologies have biases, including GC content-related biases, that affect expression measures (44). Thus, we investigated the relationship between a transcript’s GC content and its expression level as estimated from the RNA-seq data. For each bacterial system used in this study, we computed the correlation between its genes’ GC contents and its genes’ expression levels in each assayed condition. We did not observe significant correlations (*P* < 0.01) between GC contents and expression levels of transcripts based on our RNA-seq data. We did not explore more complex relationships, beyond correlation, between GC-content and expression, though it may well be the case that GC content biases are unimodal, with both high GC content and low GC content transcripts being under-represented by sequencing reads (44).

### Testing for differential gene expression

When determining whether a gene is differentially expressed in two conditions, stronger conclusions can be drawn if data are available from biological replicates. In the case when replicates are unavailable for two different conditions, the two conditions under consideration are considered surrogate replicates for each other. In this case (without true replicates), the variability between the ‘surrogate’ replicates is expected to be larger than the variability between ‘true’ replicates would be, and weaker conclusions are drawn. Rockhopper uses information from biological replicates when available. When biological replicates are not available, Rockhopper uses surrogate replicates.

Before calculating whether a gene is differentially expressed in two conditions, the variance of the gene’s expression is first estimated. The variance of a gene’s expression in a condition is initially computed, as the sample variance of the gene’s expression across the replicates. However, variance depends on the expression level. Genes with high expression levels tend to have higher variance across replicates (Supplementary Figure S3). Local regression, Lowess, is used as in Supplementary Figure S3 to obtain a smooth estimate of the variance (33).

To determine whether a gene shows differential expression in data from two conditions, we perform a statistical test for the null hypothesis, which is that the expression of the gene in the two conditions is the same. A Poisson distribution is often used to model reads from RNA-seq data (31). Although a Poisson model may be appropriate for technical replicates when variability is lower, with higher variability in biological replicates or surrogate replicates, the Poisson model does not control Type-I error and underestimates the variability, as overdispersion can be observed. Thus, following the work of others (33,45), we use the Negative Binomial distribution as our statistical model, which is more robust than the Poisson. Using this model, a two-sided *P*-value is computed indicating the probability of observing the gene’s two expression levels, in the two conditions, by chance (see ‘Materials and Methods’ section). Because multiple tests are being performed across the set of genes, *q*-values are reported that control the false discovery rate using the Benjamini–Hochberg procedure (34).

**Figure 5.**
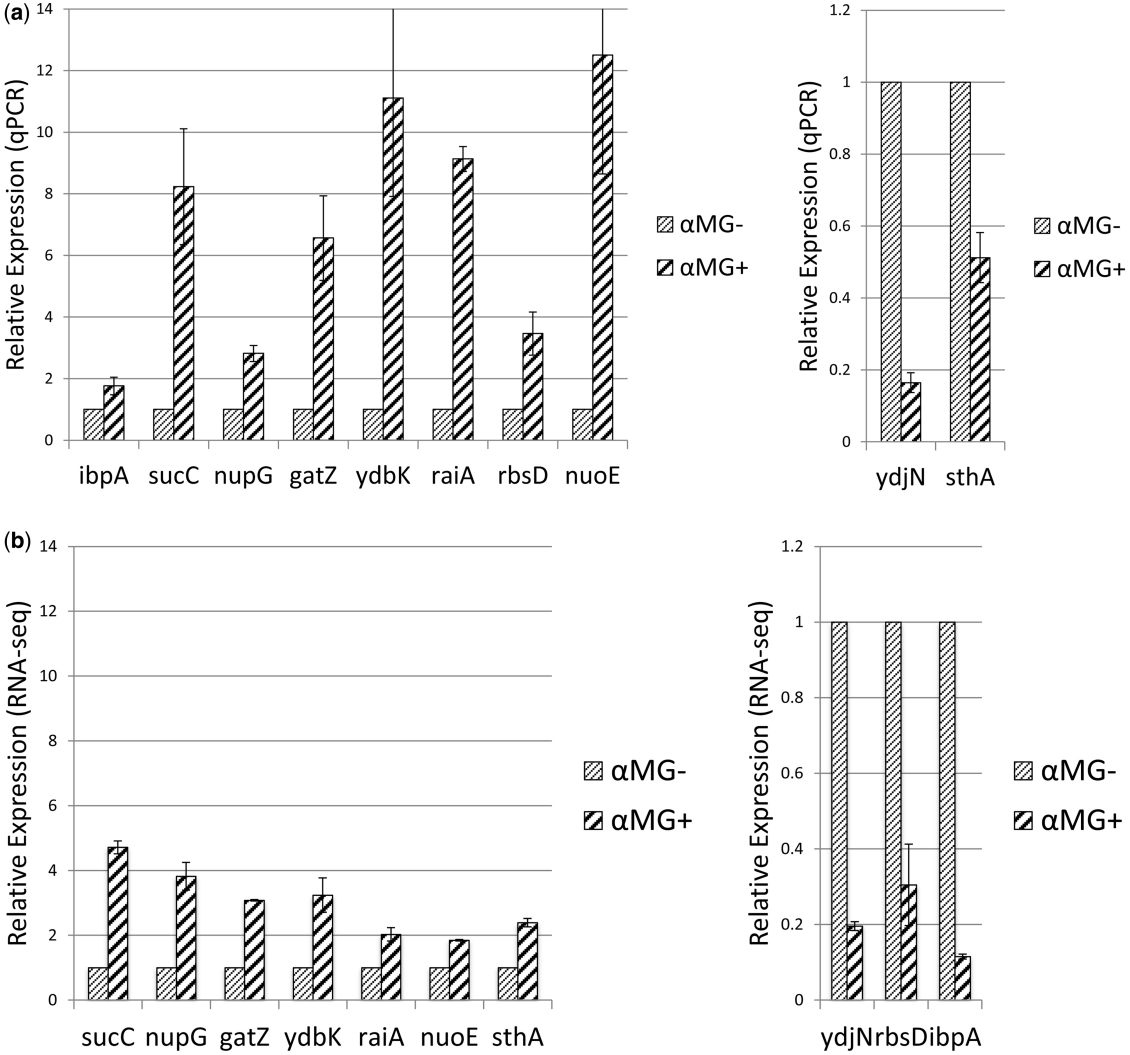
Relative expression of 10 *E. coli* genes from wild-type cells grown in LB medium with αMG as compared with expression of the same genes from cells grown in LB medium without αMG. Error bars in the figure are determined from three biological replicates. (**a**) Relative expression of the 10 genes as determined from qPCR. (**b**) Relative expression of the 10 genes as determined from RNA-seq.

To evaluate Rockhopper’s ability to identify differentially expressed genes based on RNA-seq data, we used RT-qPCR to investigate 10 randomly chosen *E. coli* genes that Rockhopper identified as differentially expressed. For each of the 10 *E. coli* genes, we looked at the change in transcript levels in wild-type cells grown in LB medium with versus without addition of the glucose analog αMG. Three biological replicates were used both for the RNA-seq experiments and the RT-qPCR experiments. Figure 5a shows the relative expression of each gene in the two conditions as determined by RT-qPCR. Figure 5b shows the relative expression of each gene in the two conditions as estimated from the RNA-seq data. Six of the seven genes (*sucC*, *nupG*, *gatZ*, *ydbK*, *raiA* and *nuoE*) estimated to be upregulated on αMG addition based on the RNA-seq data were corroborated by the RT-qPCR data. One of the three genes (*ydjN*) estimated to be downregulated by αMG addition based on the RNA-seq data was corroborated by the RT-qPCR data. Though the direction of regulation determined from the RNA-seq data was confirmed by RT-qPCR for 7 of the 10 genes, the fold change determined from the RNA-seq data was generally underestimated. For 3 of the 10 genes (*sthA*, *rbsD* and *ibpA*), the direction of regulation determined from the RNA-seq data disagreed with that determined by RT-qPCR.

For the three genes where the direction of regulation determined from RNA-seq data disagreed with the direction evinced by RT-qPCR, we investigated whether the disagreement was likely a result of the computational analysis or of the underlying RNA-seq data. For *rbsD* and *ibpA*, all alignment tools used in this study (Rockhopper, Bowtie, Bowtie2, SOAP2, BWA) mapped fewer RNA-seq reads to the gene in the +αMG condition than in the −αMG condition, even though the RT-qPCR data suggest that these two genes are upregulated in +αMG relative to −αMG. Similarly, for *sthA*, all alignment tools used in this study mapped more RNA-seq reads to the gene in the +αMG condition than in the −αMG condition, even though the RT-qPCR data suggest that this gene is downregulated in +αMG relative to −αMG. As evinced by the error bars in Figure 5, expression of the three genes was consistent across biological replicates within an experimental approach (RNA-seq or RT-qPCR), though the results differed substantially between experimental approaches (RNA-seq or RT-qPCR). These findings indicate that the disagreement between the RNA-seq regulation estimates and the RT-qPCR determined regulation for these three genes is not likely to be an artifact of the computational analysis but rather a discrepancy between the expression of the genes as assayed by the underlying RNA-seq process and the RT-qPCR process.

### Operon prediction

Multi-gene operons are a means by which bacteria can implement co-expression of related genes, and systematic identification of operons is an important step toward reconstruction of regulatory networks. Numerous computational approaches have been proposed, based on various features suggestive of polycistronic messages, for predicting operon structures throughout a genome [reviewed in (46)]. When experimental data are unavailable for elucidation of global operon maps, computational methods can use a number of features in an attempt to predict the likelihood of co-transcription for consecutive genes on the same strand, including but not limited to intergenic distance, codon usage similarity, interspecies conservation and similarity of functional annotation. Of these non-experimental features, intergenic distance between consecutive genes on the same strand has the greatest predictive power (35,47,48). High-throughput experimental data have also been used extensively to determine global operon maps, based on both microarray experiments (47,49–51) and RNA-seq experiments (9,52–55).

We investigated the extent to which operon structures can be predicted based on RNA-seq data. We used two features to estimate the probability that consecutive genes on the same strand are co-transcribed: the distance in nucleotides between the genes and the similarity of the genes’ expression in the RNA-seq data. The features are combined, together with a prior probability, using a naïve Bayes classifier to determine the probability of co-transcription (see ‘Materials and Methods’ section).

To evaluate the accuracy of our operon predictions based on RNA-seq data, we first compared the predictions with experimentally confirmed operons in *E. coli* as reported in RegulonDB (56). Based on our *E. coli* RNA-seq data, we predicted 838 multi-gene operons in *E. coli* and RegulonDB contains 843 multi-gene operons. We found that 90% of gene-pairs reported to be co-transcribed in RegulonDB were predicted to be co-transcribed by our approach, and 81% of gene-pairs on the same strand reported not to be co-transcribed in RegulonDB were predicted not to be co-transcribed by our approach (Table 1). We then investigated the extent to which our operon predictions based on RNA-seq data corresponded to predictions from a leading computational approach for operon prediction (48), whose predictions are available from the Database of prokaryotic operons (DOOR) database (57). We generated operon predictions for four different genomes using RNA-seq data and compared the results with predictions from the DOOR database. We found that our predictions corresponded to those found in DOOR with sensitivities ranging from 89 to 96% and specificities ranging from 86 to 95% (Table 1).

**Table 1.** Summary of the accuracy of Rockhopper’s operon identifications

Genome	Database	Number of multi-gene operon predictions from Rockhopper	Sensitivity (%)	Specificity (%)
*E. coli*	RegulonDB	838	90	81
*E. coli*	DOOR	838	95	89
*N. gonorrhoeae*	DOOR	410	89	87
*S. pyogenes*	DOOR	412	96	95
*S. enterica*	DOOR	868	92	86

The table indicates how operon predictions from Rockhopper based on RNA-seq data correspond to experimentally confirmed operons in *E. coli* listed in the RegulonDB database and how well operon predictions from Rockhopper correspond to computationally predicted operons in the DOOR database.

Sensitivity refers to the fraction of consecutive gene pairs on the same strand and reported to be co-transcribed in the database that are predicted as co-transcribed by Rockhopper.

Specificity refers to the fraction of consecutive gene pairs on the same strand and reported not to be co-transcribed in the database that are not predicted as co-transcribed by Rockhopper.

Operon predictions from *X. nematophila* RNA-seq data were not included because *X. nematophila* is not included in the DOOR database and because all *X. nematophila* RNA-seq experiments in this study were conducted on size-selected RNA.

**Figure 6.**
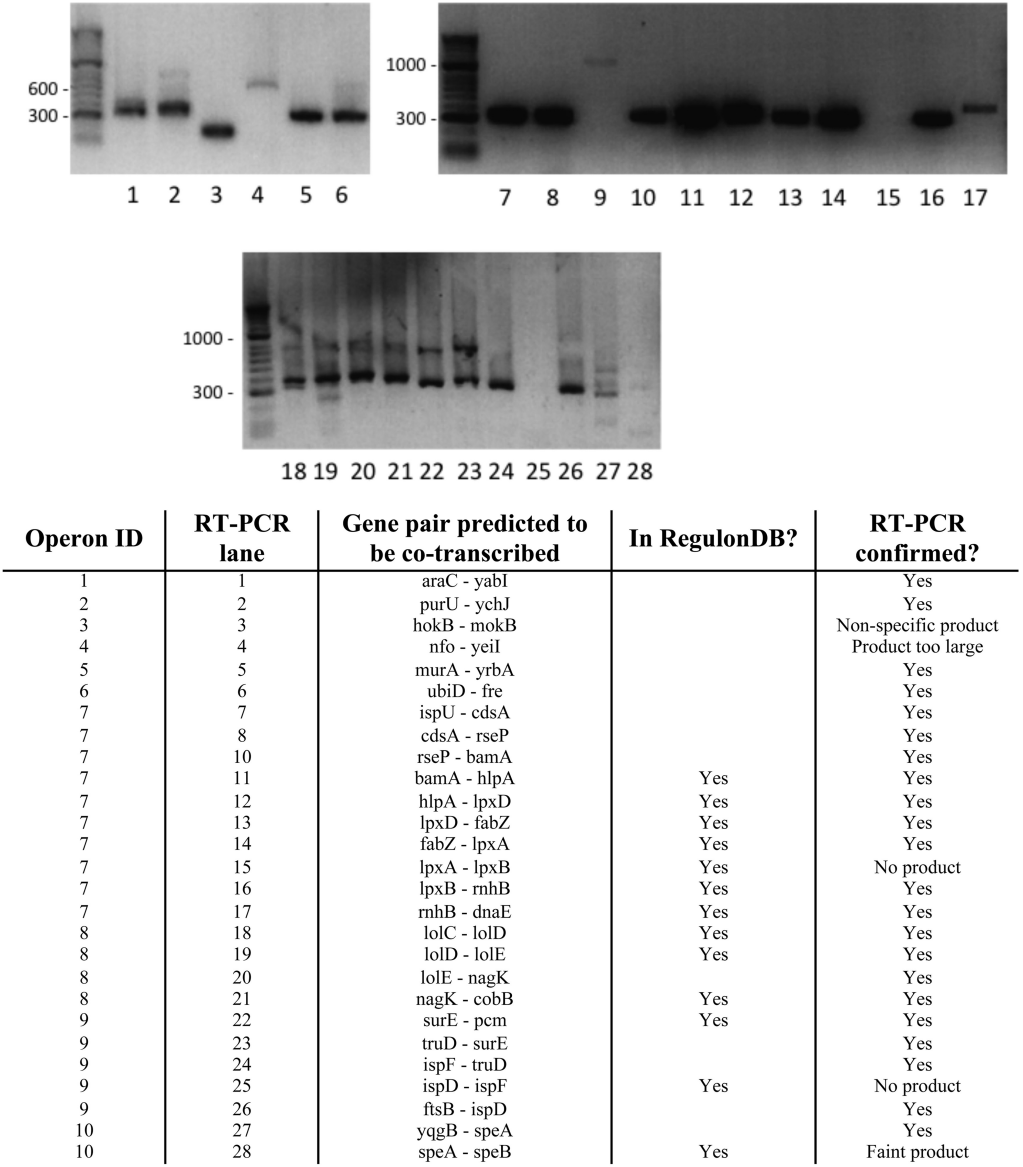
RT-PCR results for pairs of genes predicted to be co-transcribed. Lanes 9 and 10 in the RT-PCR figure correspond to two different promoters for the *rseP-bamA* operon. The 27 assayed pairs of genes correspond to 10 predicted operons containing 13 pairs of genes that were previously shown to be co-transcribed and containing 14 pairs of genes not previously shown to be co-transcribed.

In the aforementioned analysis, we classified a prediction from our system as a false positive if the predicted operon was not found in the database. However, some of these predictions might not actually be false positives, but rather operons that have not been confirmed previously or were not included in the database for some other reason. To investigate our possible false positive predictions more carefully, we chose 10 random sets of genes in *E. coli* that were significantly expressed and that we predicted, based on the RNA-seq data, to be co-transcribed as part of 10 multi-gene operons, though each set of 10 genes contained genes that were not identified as being part of an operon in the RegulonDB database. The 10 sets of genes (predicted operons) contained different numbers of genes, ranging from 2 to 11. For the 10 sets of genes, we used RT-PCR to test for co-transcription of 13 pairs of genes that were reported to be co-transcribed in RegulonDB and of 14 pairs of genes that were not reported to be co-transcribed in RegulonDB (Figure 6). The RT-PCR experiments showed evidence of co-transcription for 11 of the 13 pairs of genes that were reported to be co-transcribed in RegulonDB (Figure 6). The RT-PCR experiments showed evidence of co-transcription for 12 of the 14 pairs of genes that we predicted to be co-transcribed but were not included in RegulonDB (Figure 6). These results suggest that many of our operon predictions that we classified as false positives may correspond to operons and that the RegulonDB database is not complete. Thus, the specificity of our operon predictions is likely to be significantly higher than what we report based on our database comparison.

**Figure 7.**
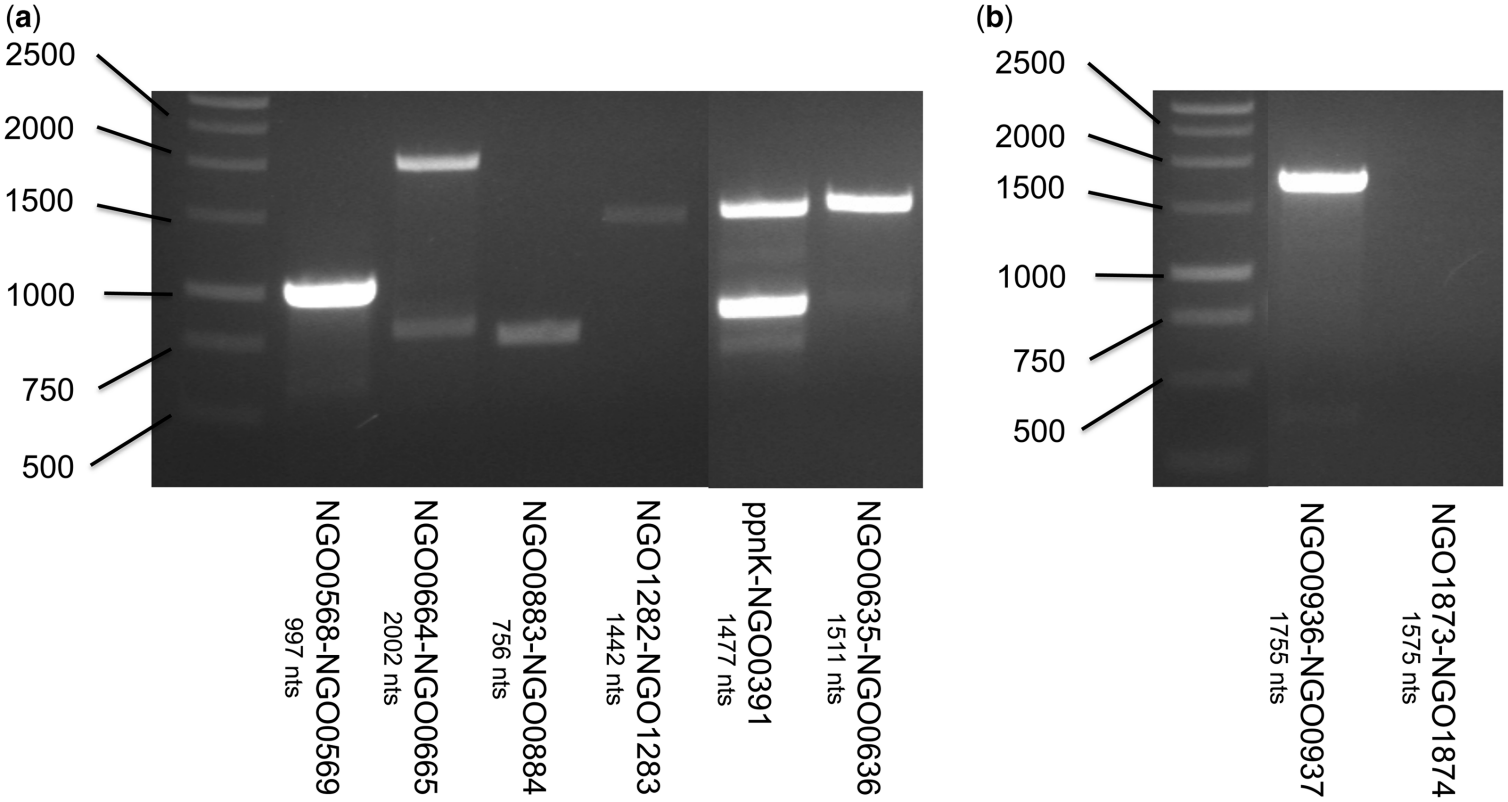
RT-PCR analysis of pairs of consecutive genes from *N.gonorrhoeae* F62 wild-type bacteria. RT-PCR was performed on total RNA by using primer pairs designed to span the entire region containing gene pairs. Below each lane, the gene pair is listed along with the size of the region containing the gene pair. (**a**) RT amplification products are evident for six gene pairs predicted to be co-transcribed by Rockhopper based on RNA-seq data. (**b**) RT amplification products are evident for one of two gene pairs predicted *not* to be co-transcribed by Rockhopper based on RNA-seq data.

Based on the high accuracy of our method in distinguishing co-transcribed genes from genes that are not co-transcribed, as determined earlier in the text, we proceeded to test with RT-PCR a small subset of our operon predictions in *N. gonorrhoeae*, where few operons have been experimentally characterized. All tested operons contained one or more genes annotated as hypothetical proteins so that there was no *a priori* annotation bias suggestive of operons. We performed RT-PCR experiments on six pairs of genes that we predicted to be co-transcribed based on our RNA-seq data, and we found evidence of co-transcription for all six pairs of genes (Figure 7a). We then performed RT-PCR experiments on two pairs of genes that we predicted not to be co-transcribed based on our RNA-seq data, and we found evidence of co-transcription for one of the two pairs of genes (Figure 7b). We found the high sensitivity (six/six) and modest specificity (one/two) of our experimentally tested predictions to be encouraging, particularly as the predictions were based on RNA-seq experiments from only two conditions. We did not explore the possibility of genes being co-transcribed in some conditions and not others, though such flexibility in operon structures may not be uncommon (9,51).

### Visualization of results

Results from Rockhopper are displayed using the Integrative Genomics Viewer (58) to enable visualization and exploration. Rockhopper generates tracks in the genome browser corresponding to reads aligned to the genome, differentially expressed genes, UTRs, novel transcripts and operons (Supplementary Figure S4). As data from each RNA-seq experiment are normalized, results from different experiments can be viewed simultaneously and meaningfully compared.

## DISCUSSION

RNA-seq experiments have proven to be a powerful approach for assaying bacterial transcriptomes. However, analysis of the large resulting data sets can be a limiting factor for studies using RNA-seq experiments. There are numerous tools available that support various aspects of RNA-seq data processing, management and analysis, but typically for a single study using RNA-seq experiments, a variety of tools must be used, often in a piecemeal fashion and possibly requiring the user to possess computational expertise. Importantly, there is a paucity of tools designed specifically for *bacterial* RNA-seq data analysis. Although tools for analysis of eukaryotic RNA-seq data are often applied to bacterial RNA-seq data, the tools are limited by their lack of appropriate modeling of bacterial gene structures and transcriptomes, including operon structures, sRNAs and dense genomes with overlapping genes.

Here, we present a set of algorithms for analysis of bacterial RNA-seq data, implemented in a user-friendly open-source software tool named Rockhopper. We evaluated Rockhopper on >2 billion reads from 75 RNA-seq experiments conducted using five different bacteria. Our motivation for using a range of RNA-seq experiments is to help us understand the robustness of our methods when applied to RNA-seq data from experiments using different bacteria under disparate experimental conditions. When aligning reads to a bacterial genome, we found that Rockhopper had comparable or higher accuracy than the four leading tools that we evaluated.

Rockhopper uses a novel approach for constructing a transcriptome map from bacterial RNA-seq data, which we found to have high precision. UTRs identified by Rockhopper generally corresponded well with primer extension and RACE results. The transcriptome map that we constructed for *N. gonorrhoeae* included 34 small transcripts that we identified as candidate sRNAs. Eleven of these candidate sRNAs were evaluated in another study by comparison to the Rfam database (41) and by northern analysis and were found to correspond to small transcripts of the expected size. These 11 small transcripts include five antisense to protein-coding genes and six in intergenic regions between protein-coding genes. Currently, when generating a transcriptome map, Rockhopper seeds the map based on any annotated genes. In future work, we plan to remove this seed requirement so that Rockhopper can be used to assemble transcriptome maps when no gene annotations are available, such as in many metatranscriptome studies.

Rockhopper also estimates transcript abundances and tests for differential gene expression between experimental conditions. We identified 1827 genes in *E. coli* evincing statistically significant differential expression in 16 conditions assayed by RNA-seq experiments. We found gene expression levels estimated by Rockhopper to have a correlation of 0.55 with levels determined by qRT-PCR in *N. gonorrhoeae*. The modest correlation can be explained, at least in part, by the experimental design of the *N. gonorrhoeae* RNA-seq experiments. The *N. gonorrhoeae* RNA-seq experiments, in contrast to our RNA-seq experiments in other bacteria, generated a shorter read length (36 nt as compared with 40–100 nt), yielded reads with a lower average quality score (31 as compared with 35) and resulted in fewer reads on average being aligned to the genome (55% as compared with 80%). In *E. coli*, for 7 of 10 genes examined, Rockhopper’s differential expression estimates agreed with those from RT-qPCR data. For the 3 of 10 genes that disagreed, our investigations suggest that the disagreement stems not from the statistical test for differential expression but from a discrepancy between the RNA-seq data and the RT-qPCR data. Interestingly, when we used randomly chosen subsets of reads from our *N. gonorrhoeae* RNA-seq experiments, above about a million reads of length 36 nt each for a 2.2 megabase genome, we did not find correlation between qRT-PCR expression levels and those estimated by Rockhopper to change significantly. These results provide a small window of insight into the depth of RNA-seq coverage necessary so as not to compromise on the accuracy of gene expression estimates. Indeed, on an Illumina HiSeq 2000 system, we observed no degradation in read quality or in downstream analyses of read data when multiplexing up to eight samples per lane, resulting in >200 million reads per lane and ∼27 million reads per sample. We did not try multiplexing more than eight samples per lane. These findings suggest that when cost of sequencing is a constraint, multiplexing large numbers of samples may be an option, though it results in fewer reads per sample, it may not significantly impact the accuracy or reproducibility of analyses of the data.

Operons are a means by which multiple genes can be controlled by a single regulatory system in bacteria. Understanding which sets of genes are co-transcribed can help illuminate regulatory networks. We describe a novel approach for characterizing bacterial operon structures based on RNA-seq data. We demonstrated that this approach, using *E.coli* RNA-seq data from 16 conditions, was able to characterize 90% of experimentally confirmed operons in *E.coli*, and we confirmed via RT-PCR six previously uncharacterized operons that were identified by our approach using RNA-seq data from *N.gonorrhoeae* and 12 previously uncharacterized operons that were identified by our approach using RNA-seq data from *E.coli*. We did not investigate Rockhopper’s ability to discern operons controlled by multiple promoters, though we expect instances of such operons to be increasingly revealed as the use of bacterial RNA-seq experiments grows.

Computational methods for analysis of bacterial RNA-seq data need to keep pace with the increasing use of RNA-seq experiments that assay bacterial transcriptomes. The large data sets that result from RNA-seq experiments necessitate systematic analyses that are both accurate and reproducible. The Rockhopper system is one attempt at such a set of systematic analyses for bacterial RNA-seq data. Rockhopper is integrated with a genome browser to enable exploration of RNA-seq data and visualization of analysis results. Rockhopper is designed as a first step following an RNA-seq experiment so as to facilitate investigation of the myriad downstream questions that bacterial researchers may wish to explore.

## SUPPLEMENTARY DATA

Supplementary Data are available at NAR Online: Supplementary Tables 1–8, Supplementary Figures 1–4 and Supplementary References [23,59,60].

## FUNDING

National Science Foundation (NSF) [MCB0919808 to B.T.] and the National Institutes of Health [R01AI048611 to C.G. and R.M.]. Funding for open access charge: NSF grant [MCB0919808 to B.T.].

*Conflict of interest statement*. None declared.

## Supplementary Material

Supplementary Data
