# Supplementary material for: Computational analysis of bacterial RNA-Seq data
Source: Nucleic Acids Res. 2013 May 28;41(14):e140. doi: 10.1093/nar/gkt444 (PMC3737546; doi:10.1093/nar/gkt444)
Supplement: Supplementary Data [file supp_41_14_e140__index.html]

Computational analysis of bacterial RNA-Seq data — Computational analysis of bacterial RNA-Seq data — Supplementary Data 

# Computational analysis of bacterial RNA-Seq data

## Supplementary Data

files

**Files in this Data Supplement:**

- Supplementary Data - pdf file
- Supplementary Data - xlsx file
