# Supplementary material for: Computational analysis of bacterial RNA-Seq data
Source: Nucleic Acids Res. 2013 May 28;41(14):e140. doi: 10.1093/nar/gkt444 (PMC3737546; doi:10.1093/nar/gkt444)
Supplement: Supplementary Data [file supp_gkt444_nar-00089-met-k-2013-File010.pdf]

## Supplementary Figures (S1 - S4) for

## Computational Analysis of Bacterial RNA-Seq Data

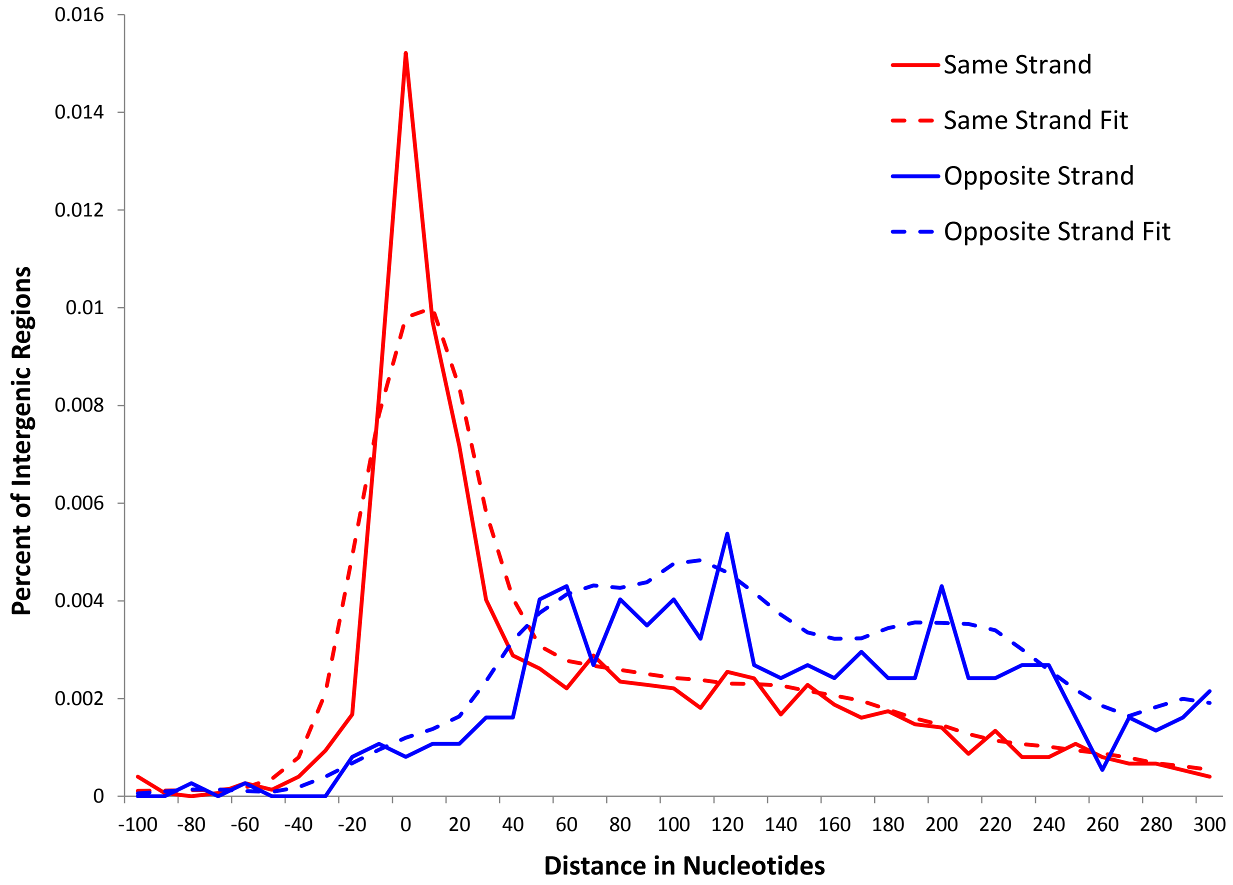


**Figure S1:** Distribution of distances between consecutive genes. Distributions of distances between consecutive genes on the same strand (solid red line) and between consecutive genes on the opposite strand (solid blue line) in *Streptococcus pyogenes* are shown. Distances are negative when consecutive genes overlap. The dashed lines are density functions estimated from the empirical distributions using an Epanechnikov kernel.

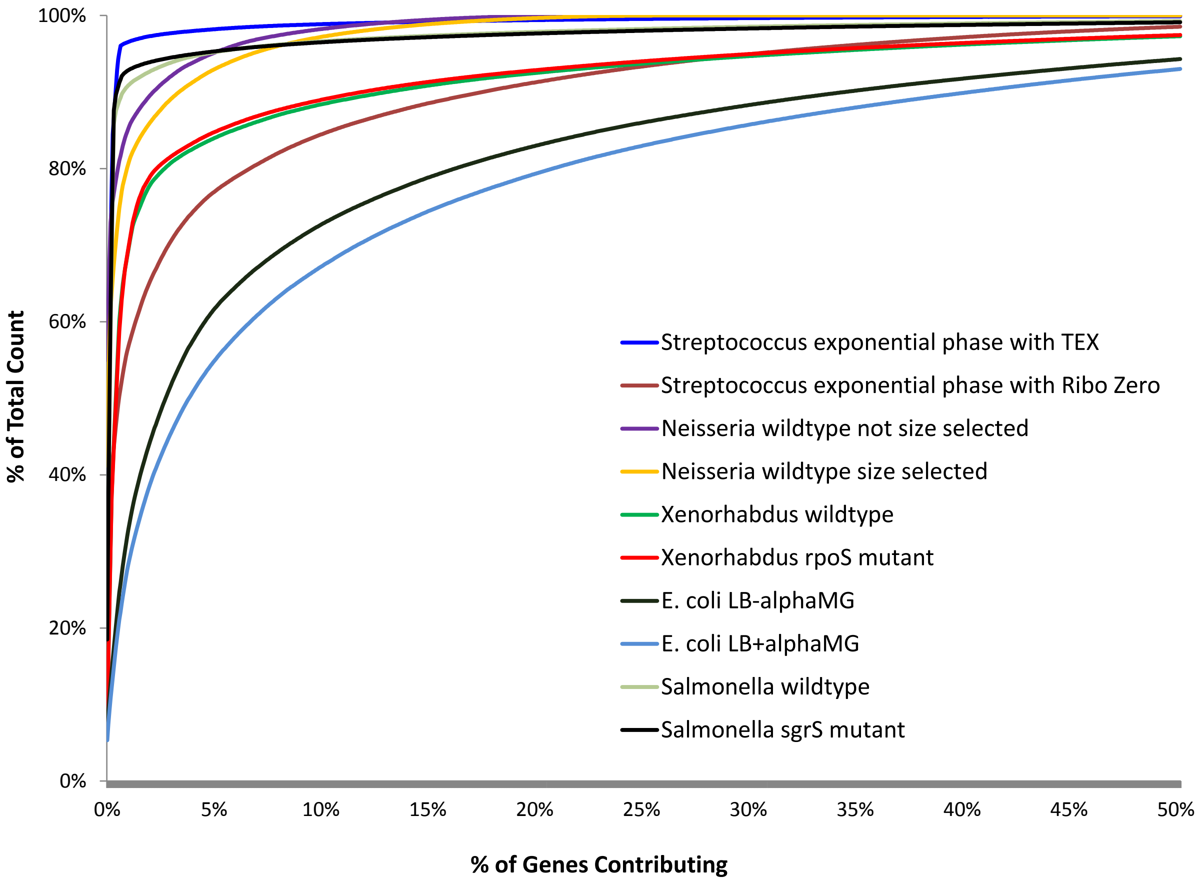


**Figure S2:** Cumulative percentage of read counts. The cumulative percentage of total read counts from ten samples is shown, beginning with the most highly expressed gene. The samples correspond to RNA-seq experiments, two each, performed using five different bacteria. All ten samples were depleted for ribosomal RNA prior to sequencing. The 5% of most expressed genes account for between 55% and 98% of total read counts in the ten samples.

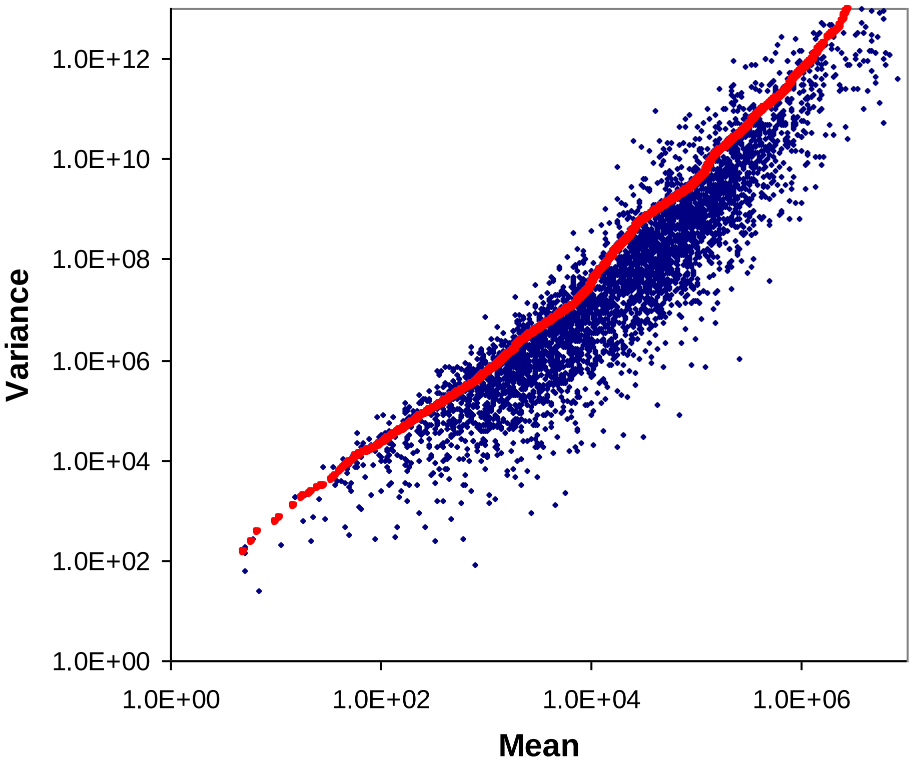


**Figure S3:** Relationship between a gene's expression level and variance. Each point in the log-log scatter plot represents a gene's expression level vs. the gene's expression variance as determined from RNA-seq experiments conducting using *Salmonella*. The red line is the computed Lowess fit.

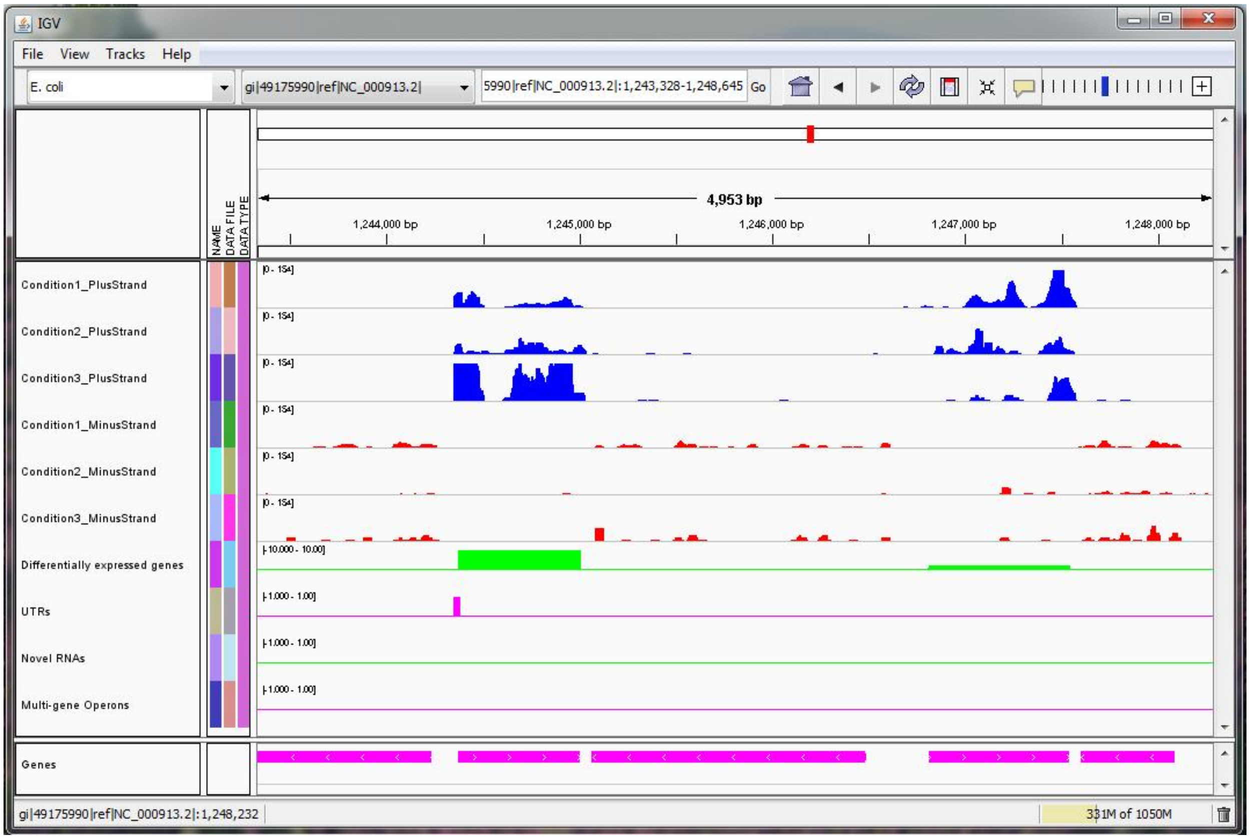


**Figure S4:** Results from Rockhopper displayed in the IGV genome browser. The blue and red tracks correspond to sequencing reads, in each of three experiments, that Rockhopper aligned to the plus strand and to the minus strand of the genome, respectively. One green track corresponds to differentially expressed genes, with height of green bars indicative of significance of differential expression. Two genes on the plus strand are differentially expressed in the displayed region. The second green track corresponds to novel RNA transcripts. There are no novel RNA transcripts in the displayed region. One purple track corresponds to UTRs of protein coding genes, as identified by Rockhopper based on the sequencing data. There is one 5'UTR on the plus strand in the displayed region. The second purple track corresponds to multi-gene operons identified by Rockhopper. There are no multi-gene operons in the displayed region. The final purple track at the bottom of the image corresponds to protein coding genes and RNA genes annotated in RefSeq.
